# An Investigation to Identify the Anti-Cancer Properties of Novel Rhenium Compounds in Prostate and Lung Cancer Cell Lines

**DOI:** 10.26502/jbb.2642-91280206

**Published:** 2026-02-04

**Authors:** Christopher Krauss, Jazmine Cuffee, Narendra Banerjee, Erik Armstrong, Satyendra Banerjee, Somiranjan Ghosh, Tanmoy Mondal, Zahidur Abedin, Santosh Mandal, Hirendra Nath Banerjee

**Affiliations:** 1 Department of Natural Sciences, Pharmaceutical Science, Computer Science, Engineering & Technology, Elizabeth City State University Campus of the University of North Carolina, Elizabeth City, 27909, USA; 2 Department of Biology, Howard University, Washington DC, USA; 3 Departments of Pediatrics and Child Health, College of Medicine, Howard University, Washington DC, USA,; 4 PrimBio Research Institute, Garnet Valley, Pennsylvania, USA; 5 Department of Chemistry, Morgan State University, Baltimore, MD, USA

**Keywords:** Apoptosis, Rhenium Compounds, Tubulin, IPA

## Abstract

The anti-cancer properties of rhenium compounds have attracted researchers for decades. In this study, rhenium-based PR series compounds (PR1–PR10) were exposed to LNCaP and E006AA prostate cancer cell lines to verify their toxicities. Among the compounds, PR7 was tested further to determine its effects on the lung cancer cell lines A549 using RNA sequencing and Ingenuity Pathway Analysis (IPA) software. In this study, IPA analysis was explored further to determine that PR7 played a role in various canonical pathways. The pathways affected are: *Ephrin Receptor Signaling, TGF-β Signaling, STAT3 Pathway, EGF Signaling, Tumor Microenvironment Pathway, BAG2 Signaling Pathway, H-17A Signaling Pathway, EIF2 Signaling*. Annexin-V and Tubulin tracker kits identified the apoptotic capabilities of the PR series compounds

## Introduction

The anticancer properties of rhenium are of importance because of its non-toxic behavior towards normal cells [1–4]. With existing anti-cancer medications starting to see issues with cancer resistance, the use of new anti-cancer agents such as rhenium compounds is becoming increasingly important [5]. While many researchers have demonstrated the anti-cancer properties of rhenium compounds, there has not been many publications how rhenium compounds achieve these results.

Tubulin is a protein whose functions in the cell cycle are well established [6]. Cancer cells are able to alter tubulin for its own benefit to enhance its aggressiveness and to improve resistance to treatment [6]. By disrupting the tubulin-microtubule equilibrium, either by stabilization or destabilization, cell-cycle arrest can be triggered and results in apoptosis [7, 8]. This occurs either by increasing the microtubule polymer mass in the case of microtubule stabilization or decreasing the polymer mass due to destabilization. Unfortunately, targeting tubulin alone has yielded limited success due to dose limiting toxicity and drug resistance [9, 10]. Rhenium has been shown to target tubulin and is not highly toxic, so it may overcome this challenge [11, 12].

Previously, rhenium-based compounds were found to play a role in tubulin dynamics from our research, but that was with a different set of compounds. To determine if there is potential for the tubulin dynamics to be caused by rhenium, this set of compounds was also tested to see if tubulin polymerization would occur. The tubulin polymerization could be used to explain the anti-cancer properties of rhenium by triggering the induction of apoptosis.

Current study was an attempt to determine the mechanism behind rhenium-based compounds with anti-cancer benefits. We investigated the genetic changes caused by exposure to a rhenium-based compound in addition to looking into the tubulin polymerization caused by exposure to a rhenium-based compound. While we previously published findings with IPA on these compounds, there was more information that could be gleaned from the data. For that reason, we decided to investigate further to see if we could find more genetic changes that could explain some of rhenium’s anti-cancer properties.

## Results

### Cell Viability Studies

**Figure 1:**
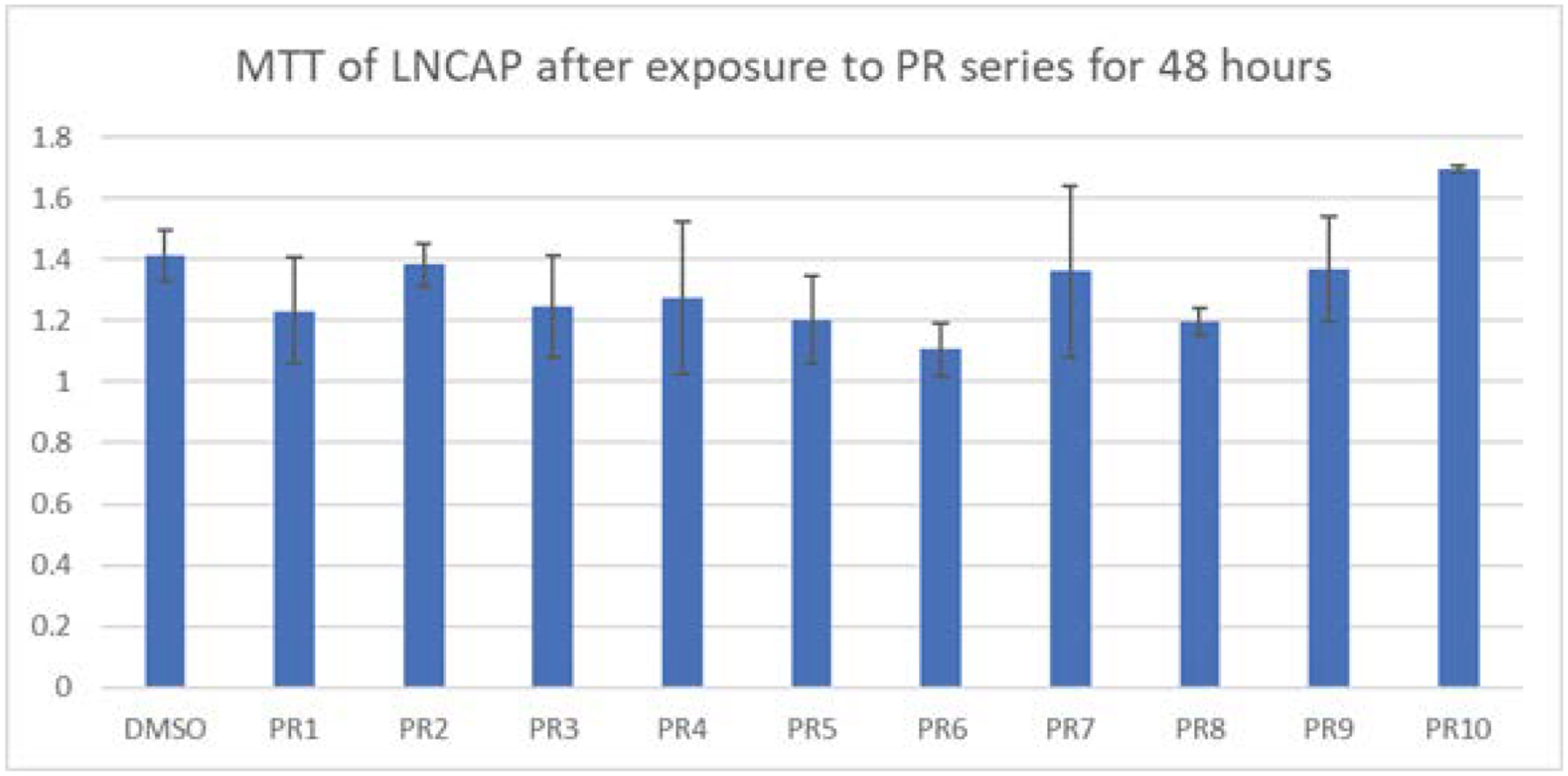
MTT of LNCaP cancer cell lines exposed to the PR series for 48 hours. LNCaP cancer cell lines were treated with 1μM of the PR series and cell viability was measured after the 48-hours. A slight decline in viability was observed with PR1, PR3, PR4, PR7, and PR9. PR5, PR6, and PR8 produced the best results. PR10 appeared to give the LNCaP a survival advantage.

Exposure of LNCaP cells to MTT showed mixed results (Figure 1). PR2 had very little decrease in viability compared to the control, while PR10 showed an improved viability compared to the DMSO control. PR1, PR3, PR4, PR5, PR6, and PR8 showed the best declines in cell viability in the LNCaP prostate cancer cells. Using a student’s t test to calculate the p values, showed that only PR6, PR8, and PR9 produced a significant amount of cell death.

**Figure 2:**
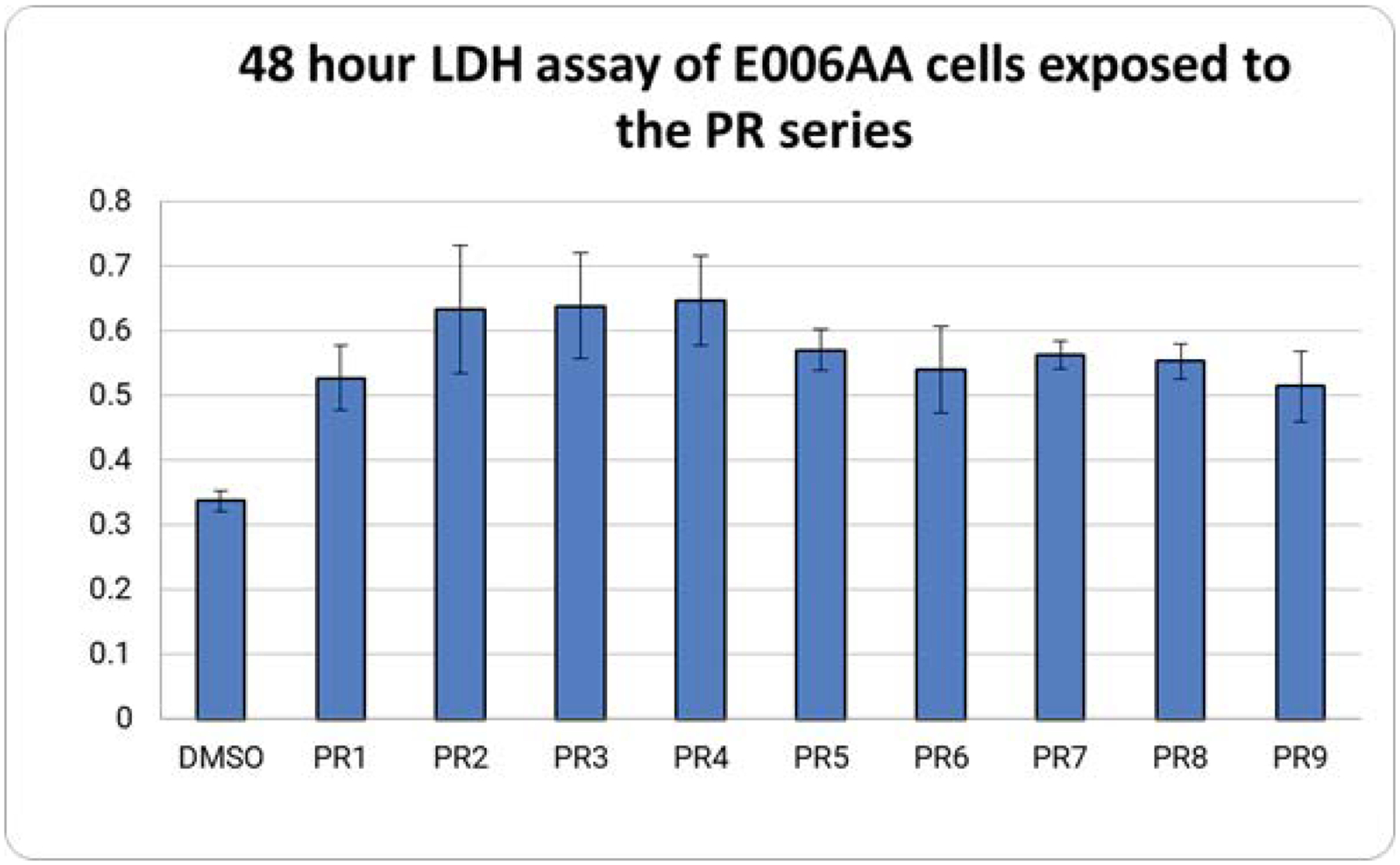
LDH assays of E006AA cells exposed to the PR series. E006AA cells were treated with 1μM of the PR series for 48 hours. More cell death was observed in the treated cells compared to the DMSO control. By the time PR10 was synthesized, ATCC had stopped selling E006AA due to complications with the cell line. Thus, the experiment could not be repeated with PR10.

Exposure of E006AA cells to the PR series produced an increase in cell death with all compounds of the PR series (Figure 2). A student’s t-test revealed that all PR compounds tested produced significant results when compared to the DMSO. Using the t value from a one-tailed student’s t test to generate p values for the PR series showed all values were under 0.05 and thus considered statistically significant.

### IPA Analysis

#### Differentially expressed genes to their biofunctions, networks, and associated diseases and disorders

The differentially expressed gene sets on biofunctions was evaluated using IPA analysis, which was based on their p-values and fold change recorded in the PR7 treated cells (Table 1). The top canonical pathways evaluated from IPA results were EIF2 Signaling Sirtuin Signaling Pathway, Regulation of eIF4 and p70S6K Signaling, BAG2 Signaling Pathway, and Ephrin Receptor Signaling. The top Molecular and Cellular Functions were Cell Cycle and Cellular Movement. Most significant tox lists were Mitochondrial Dysfunction, Liver Necrosis/Cell Death, Oxidative Stress, NRF2-meddiated Oxidative Stress Response, and Acute Renal Failure Panel (Rat). We observed, NFE2L2, ANXA2, AKT1, HDAC6 and TGFB1 were significant upstream regulator genes.

### Overall gene expression and related canonical pathways (CP) using IPA analysis

**Figure 3:**
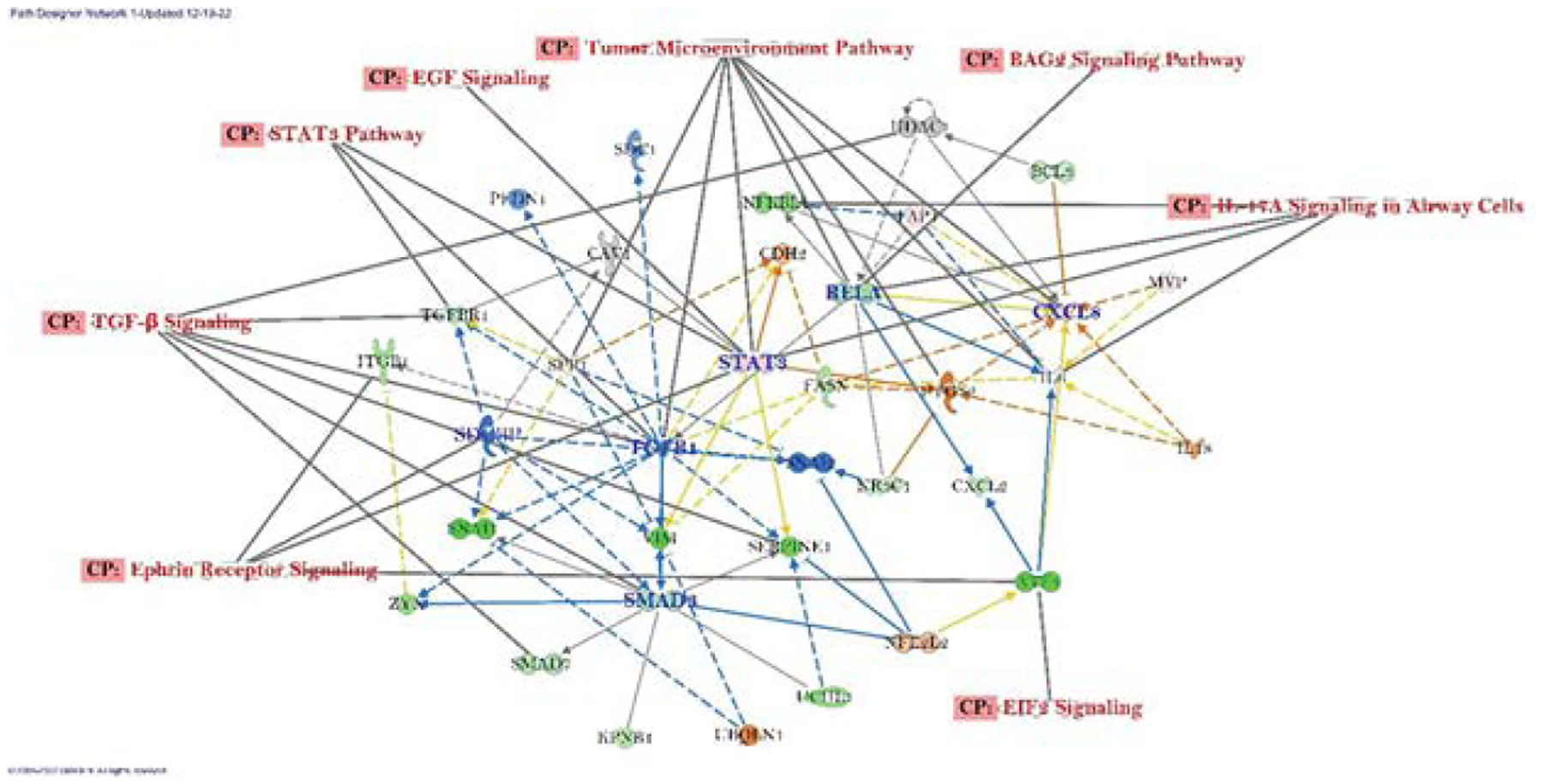
A network of connected genes whose expression was altered by PR7 in A549 lung cancer cell lines. This network contains various oncogenes that were found to be downregulated when compared to untreated A549 cell lines.

The canonical pathways with differentially expressed gene networks of connected genes whose expression was altered by PR7 in A549 lung cancer cell lines identified by IPA analysis were: *Ephrin Receptor Signaling*, TGF-*β Signaling*, STAT3 *Pathway*, *EGF Signaling, Tumor Microenvironment Pathway, BAG2 Signaling Pathway, H-17A Signaling Pathway, EIF2 Signaling* (Figure 3). The central molecules that we observed were TGF-β1, STAT3, CXCL8, RELA, SMAD3, SEBPINE1 and SDCBP out of which STAT3 and CXCL8 were upregulated, and others were downregulated. A significant amount of inhibition noted in TGF-*β1* which also identified as key molecule in the overall network.

### Gene expression status on migration of carcinoma cell and cell cycle progression of carcinoma cell line

According to differential gene expression data, genes those are involved in migration of carcinoma cell line were both up- and down regulated (28% and 30% respectively). The connected canonical pathways were Ephrin Receptor Signaling, TGF-*β* Signaling, Tumor Microenvironment Pathway, BAG2 Signaling Pathway, p53 Signaling, and EIF2 Signaling (Figure 4). We also noted role of important gene set in the cell cycle progression of carcinoma cell line were GSK3B, HOMX1, MYC, RAF1, RB1, S100A4 and TP53. All the genes were downregulated and connected with three different canonical pathways which were Cell cycle: G2/M DNA damage and checkpoint regulation, Cell cycle: G1/S checkpoint regulation, and Cell cycle regulation by BTG Family Protein (Figure 4).

### Tubulin Assay

Tubulin is a potential target for cancer therapy and was previously reported to be a potential target for rhenium based compounds [13]. To determine if the PR series could affect tubulin in cancer cells, LNCaP was exposed to PR7 for 48 hours. After this time, the cells were stained with Tubulin Tracker Deep Red and put under a fluorescent microscope using the same protocol as reported previously. It was found that there was less staining in PR7 treated cells due to Tubulin depolymerization while more staining due to DMSO treated vehicular control due to tubulin tracker binding to polymerized tubulin in the cancer cells.

### Annexin-V FITC Apoptosis Assay

**Figure 6:**
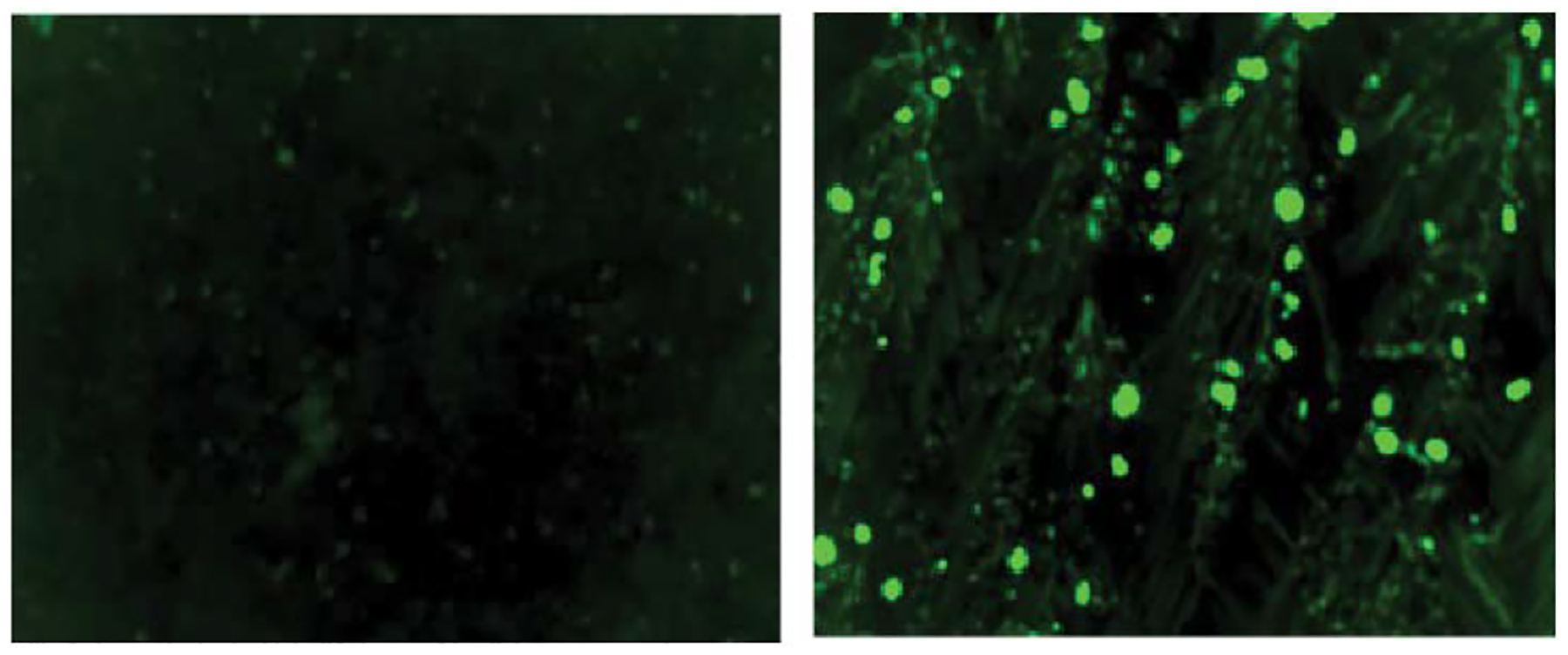
**A).** Annexin V-FITC of LNCaP exposed to DMSO for 48 hours. Lower fluorescence than the PR5 experimental sample indicates that membrane blebbing has not occurred and therefore less apoptosis is present in the control. **B).** Annexin V-FITC of LNCaP exposed to PR5 for 48 hours. A higher fluorescence in the PR5 exposed cell lines than the DMSO control is indicative of membrane blebbing and therefore the Annexin-V can bind to phosphatidylserine indicating apoptosis is occurring.

An Annexin-V FITC apoptosis assay was performed on LNCaP cell lines. PR5 was used to test for apoptosis induction in the LNCaP cells. LNCaP cells were exposed to either PR5 or DMSO for 48 hours, before staining with FITC tagged Annexin-V. The samples containing PR5 showed more fluorescence than the DMSO control (figure 6), indicating that PR5 is inducing apoptosis in the LNCaP cells.

## Discussion

Many of the genes modified by PR7 that were found to be downregulated after PR7 treatment were identified to have properties benefiting cancer cells. BCL2L1 and NQO1 are known to provide resistance to cisplatin in breast cancers [14, 15]. TRIB3, which has been linked to cancer cell migration, invasion, stemness, and EMT in lung cancers in addition to progression in breast cancers was also found to be upregulated in testicular cancer. All of these were downregulated by PR7, indicating a potential for PR7 to be used alongside cisplatin to increase cancer sensitivity to treatment. Additionally, lowering expression of TRIB3 has been proven to alleviate the advantages lung and breast cancers have gained. Alongside the inhibition of KPNB1 to induce apoptosis and the downregulation of multiple oncogenes, this suggests that PR7 could have therapeutic potential for lung and breast cancers [15, 16].

Cell viability experiments provided evidence indicating that the PR series all produced a statistically significant decline in viability in E006aa prostate cancer cells. When compared to LNCaP, only PR6, PR8, and PR9 produced statistically significant cell death. This cell death was likely due to apoptosis based on the increased fluorescence after exposure to Annexin-V FITC. The increased polymerization of tubulin and inhibition of KPNB1 also provide evidence indicating apoptosis is the likely cause of cell death due to the PR series compounds.

Alteration of tubulin dynamics can interfere with mitotic progression and result in apoptosis [8]. Either tubulin stabilization or destabilization can achieve this effect. By testing LNCaP cells exposed to PR7 compared to a DMSO control, it was discovered that the tubulin was polymerized and therefore considered to have been destabilized. This suggested that proliferation may be inhibited, and apoptosis could be induced. The Annexin-V FITC testing confirmed that apoptosis was indeed induced as evidenced by the exposure of PR5 to LNCaP cells. Unfortunately, PR5 was not tested using RNA sequencing to determine the genetic changes. However, both PR5 and PR7 contain the same rhenium core, meaning that it is likely similar genes would be influenced by both. Further testing is needed to confirm this conclusively.

While the genetic data of changes due to PR7 exposure were previously published, the publication showed only a cursory glance into the data. Review of existing literature identified potential genes associated with resistance to cisplatin, and those genes were checked using the IPA software to see if PR7 altered their expression. This search provided a few genes that were both altered by PR7 in A549 lung cancer cell lines and that are known play a role in cisplatin resistance. Two genes were found, BCL2L1 and NQO1, that are known to contribute to cisplatin resistance in breast cancer [14, 15] and TRIB3 was identified to potentially play a role in testicular cancer. PR7 decreases the expression fold change of BCL2L1 and NQO1 by −23.994 and −7.134 respectively and decreased TRIB3 expression with an expression fold change of −15.175.

IPA was also utilized to develop a network of connected molecules that were influenced by PR7 (figure 3). This information can be used to identify molecules whose changed expression via PR7 can potentially explain its anti-cancer properties. The parts of the network that are linked to the canonical pathways that we previously published were labeled in the network. Additionally, the genes identified by this network were checked to determine their significance in cancer. ITGB1, SERPINE1, CXCL2, TGFBR1, UCHL1, FASN, ATF4, and BCL3 were identified as oncogenes and were all downregulated by PR7. Additionally, it was discovered that SNAI1 and SDCBP, which are involved in the development of stem cell like properties in cancer cells, was also downregulated. TGFBR1, SNAI1, and PFDN1 all of which are involved in the process of endothelial mesenchymal transition were also found to be downregulated. Finally, KPNB1 was found to be downregulated. The downregulation of KPNB1 can be linked to an induction of apoptosis.

## Materials and Methods

### Synthesis and characterizations of the PR series compounds

The syntheses of the PR series compounds were achieved through Mandal’s Synthesis [16, 17]. First, the pentylcarbonato compounds (PC1-PC10) were synthesized from the reactions of dirhenium decacarbonyl {Re2(CO)10}, polypyridyls (aka, α-diimines) and pentyl alcohol in the presence of carbon dioxide. Second, the PC compounds were treated with perrhenic acid (HReO4) in dichloromethane. The solid PR compounds were obtained after evaporating off the solvent on a rotary evaporator. Sometimes recrystallizations of the solid PR compounds afforded crystalline PR compounds. The compounds were characterized through IR spectroscopy and in some cases through X-ray crystal structure determinations (see: C. Krauss, PhD thesis, Morgan State University, 2022).

### Cell Culture

Cells were grown in an incubator set to 37°C containing 5% CO2. Each cell line was grown in its respective growth media based on the supplier’s recommendations. A standard cell culture protocol was followed to split the cells when they reached confluence. For adherent cells, all cell culture media was removed from the cells and 2 ml of trypsin added to the flask. The flask was then returned to the incubator for 2 min. After this time, the flask was gently tapped before 2 ml of cell culture media containing fetal bovine serum was added to neutralize the trypsin. The cells were then transferred to a centrifuge and spun for 10 minutes to generate a cell pellet. The pellet was resuspended with cell culture media and put into two new flasks. The new flasks were then brought up to a total volume of 5 mL of cell culture media, before being returned to the incubator. For floating cells, the cell culture media was taken directly from the flask and put in a centrifuge tube to spin. The remaining steps for the floating cells were the same as the trypsinization process.

### MTT assay

As the PR series has different molecular weights for each of its members, we added 1 μM concentrations to each PR series per well of the 96 well plate, for the DMSO well, the highest volume of PR series needed was determined and added as vehicular control. The plates were returned to the incubator for 48 hours. After 48 hours had passed, 10 μL of MTT reagent was added to each well. The plates were then placed in the incubator for 2 hours. After this incubation, 100 μL of MTT detergent reagent was added to each well. The plate was then allowed to incubate for an additional 4 hours in the dark at room temperature. After the 4 hours incubation, the plate was read in a plate reader to determine cell viability.

### LDH assay

Cells were plated into individual wells until confluent. When all wells were confluent, the appropriate volume of PR series compounds were added to each well. For the control wells, DMSO was used as a vehicular control. The amount of DMSO added was equivalent to that of the PR series compound that needed the most volume added. After 48 hours of exposure, the cells were centrifuged, and supernatant was collected. From that supernatant, 50μL was transferred into a 96-well plate. Next 50μL of Reaction Mixture from the Pierce LDH assay kit was added to each well. The plate was then allowed to incubate at room temperature for 30 minutes in a drawer to protect the mixture from light. Finally, 50μL of stop solution was added and the plate was immediately put into a plate reader to measure the absorbance at 490nm.

### EMT Inhibition

A549 cells were cultured into 6 well plates and exposed to either 1μM PR7 or an equivalent volume of DMSO. All wells were also exposed to 2.5 ng/mL of TGF-ß to induce EMT in the cell lines. After five days of exposure, the wells were observed under a fluorescent microscope to determine if there was a decline in fluorescence due to exposure to PR7.

### Tubulin Assay

The Tubulin Tracker Deep Red kit from ThermoFisher Scientific was used for this experiment. LNCaP cells were split and grown in 6-well cell culture plates. When confluent, the cells were exposed to 1μM of PR7 for the experimental samples or an equivalent volume of DMSO for the controls. The wells were incubated for 48 hours at 37°C with 5% CO2. After the 48-hour incubation, the stock solution of Tubulin Tracker Deep Red was diluted to a 1X concentration using 1X PBS. The wells were coated with 200μL of this diluted solution and returned to the incubator for 30 minutes. After this incubation, the cells were washed using 1X PBS 3 times, before being put onto the Paula microscope to observe fluorescence.

### Annexin V-FITC Apoptosis Assay

The TACS Annexin V-FITC Apoptosis Detection Kit from R&D systems was utilized for this experiment. The cell line used for this experiment was LNCaP, and thus an adherent cell line. As apoptotic cells may begin to float, additional steps were utilized to ensure all cells were exposed to the Annexin V-FITC. First, LNCaP cells were grown in 6 well plates. Once confluent, 1μM of PR5 was added to the experimental wells and equivalent DMSO was added to control wells. The cells were returned to the incubator for 48 hours. After the 48 hours, culture media was removed, and cells were removed from the wells via trypsinization. Following trypsinization, the cells were spun in a centrifuge. The supernatant was discarded, and the cell pellet was resuspended using 1X PBS that was kept at 4°C. The cells were then centrifuged again and once again the supernatant was discarded. The collected cell pellet was resuspended in 300μL of Annexin V Incubation Reagent prepared following the kit’s protocol, omitting the optional propidium iodide. The cells were then incubated in this reagent for 15 minutes in the dark at room temperature. The cells were then centrifuged for 5 minutes, supernatant discarded, and the cell pellet was resuspended using 1X binding buffer to wash the cells. The cells were then centrifuged again before resuspended into 1X binding buffer and observed under the Paula microscope.

### RNA Isolation and RNA sequencing

RNA sequencing of A549 lung cancer cells was performed for us by PrimBio Corporation. To generate the cells for this sequencing, a plate of A549 lung cancer was exposed to TGF-ß for 10 days. Another plate of A549 was exposed to both TGF-ß and PR7 for the 10 days. These cells were collected via centrifugation and trypsinization, and the resulting pellet was mailed on ice overnight to PrimBio Corporation’s lab for RNA sequencing. This data was subsequently used in the Ingenuity Pathway Analysis study as well as to determine potential gene targets for our testing in the real time PCR study.

### Ingenuity Pathway Analysis (IPA)

Furthermore Ingenuity Pathway Analysis (IPA) software was used to find the canonical pathways that were impacted by the changes in RNA expression due to A549’s exposure to PR7 from the RNA sequencing data that we received from the servicer. Briefly data sets containing gene identifiers and their corresponding expression values (fold change) from the qRT-PCR analysis were uploaded into Ingenuity Pathway Analysis software (Ingenuity^®^ Systems, www.ingenuity.com). Each gene identifier was plotted to its similar gene entity in the Ingenuity Pathways Knowledge Base and the canonical pathway (CP) analysis which we used to build the pathways connecting the top networks. The Core analysis was performed to assess the functional association and interaction of the genes and with their respective pathways. The relationship between altered gene expression and changes in bio functions were categorized with subcategories of cellular and molecular functions, disease and disorders, and physiological system development and function respectively. Top canonical pathway analysis was investigated using specific gene functions and presented within the network connection and compared.

## Figures and Tables

**Figure 4A:**
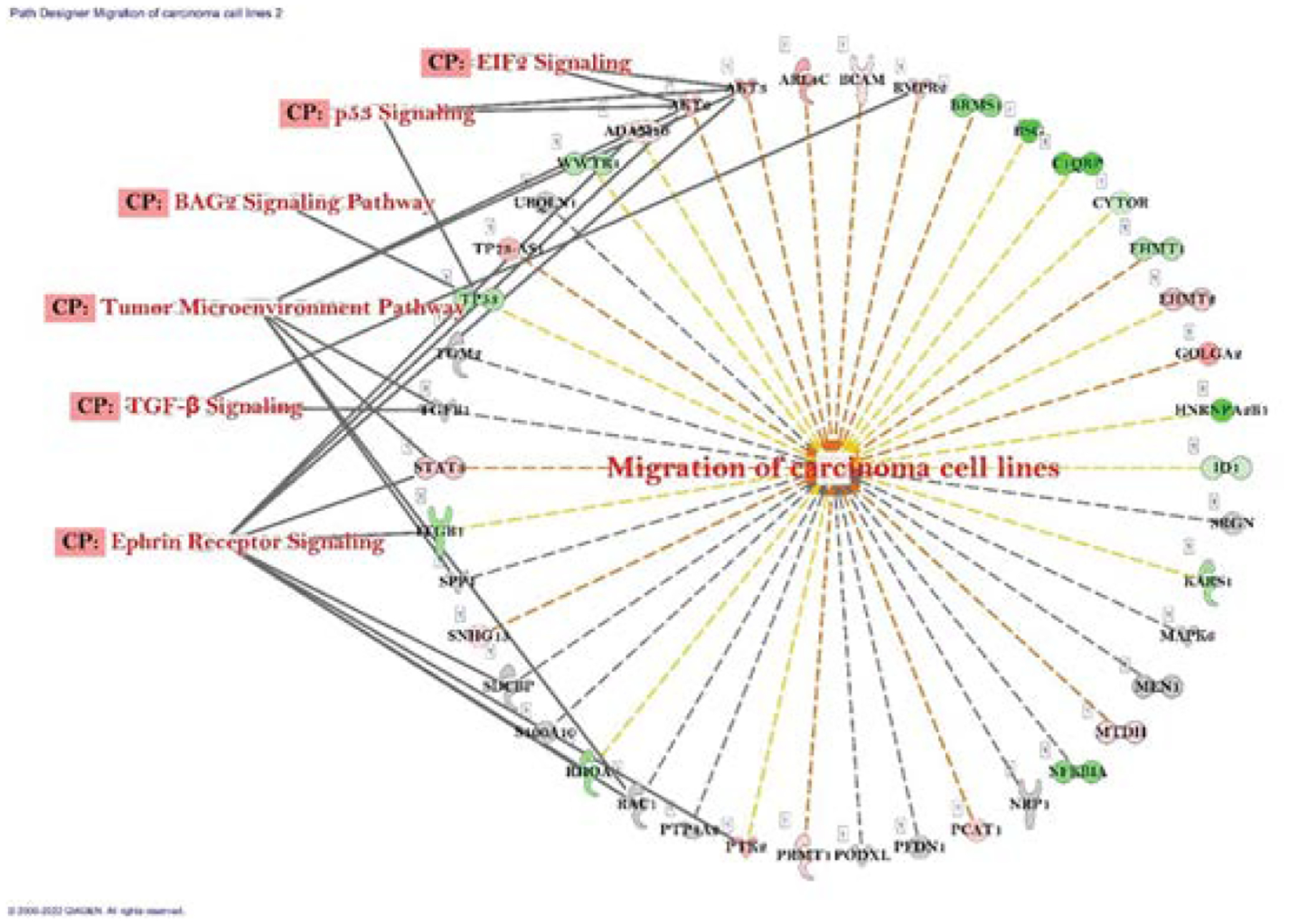
Network of differentially expressed genes and the important signaling pathways involved in migration of carcinoma cell lines after PR7 treated A549 lung cancer cells.

**Figure 4B:**
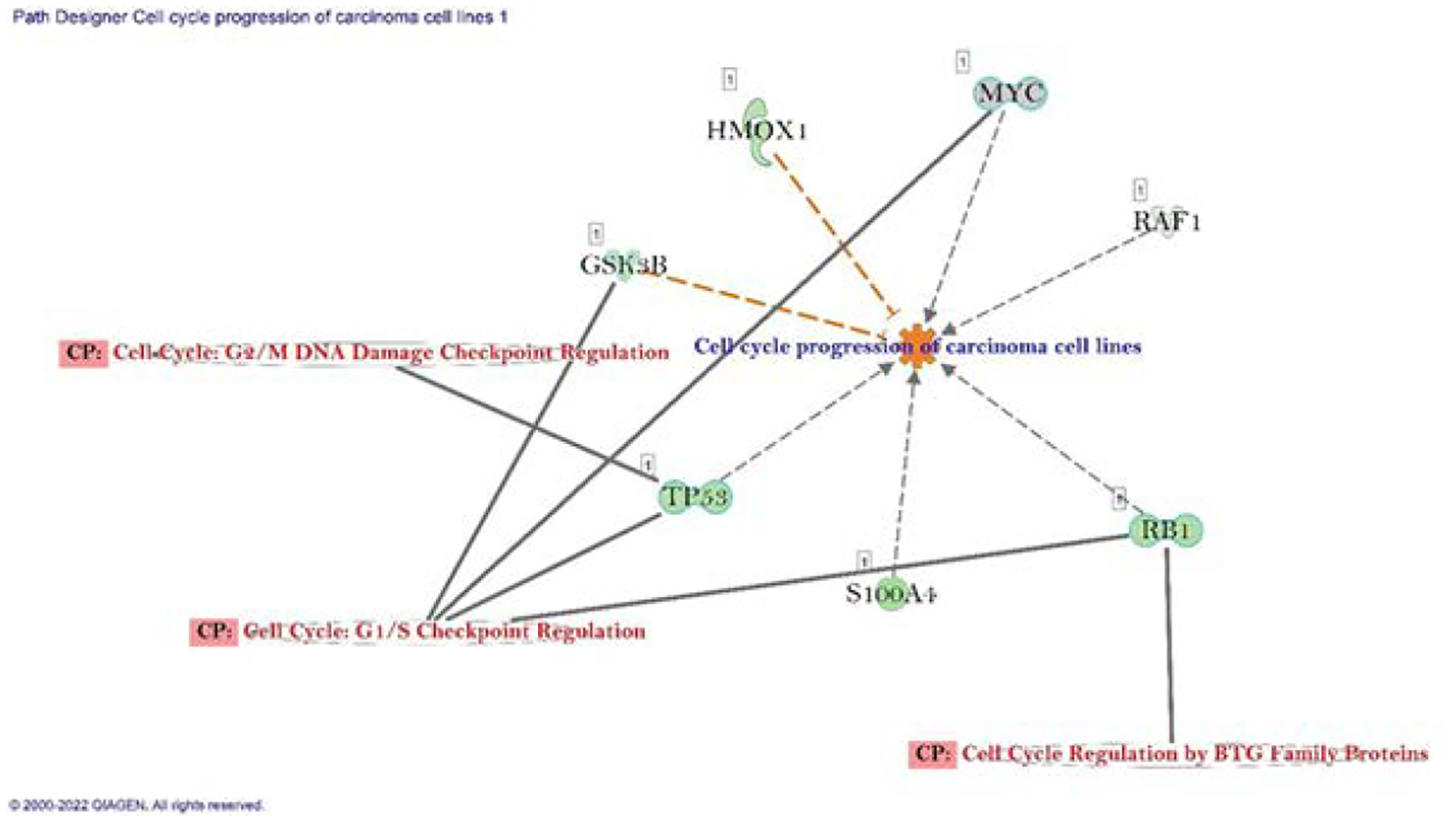
Network of differentially expressed genes in the important signaling pathways involved in cell cycle progression of carcinoma cell line in A549 lung cancer cells (PR7 treated).

**Figure 5:**
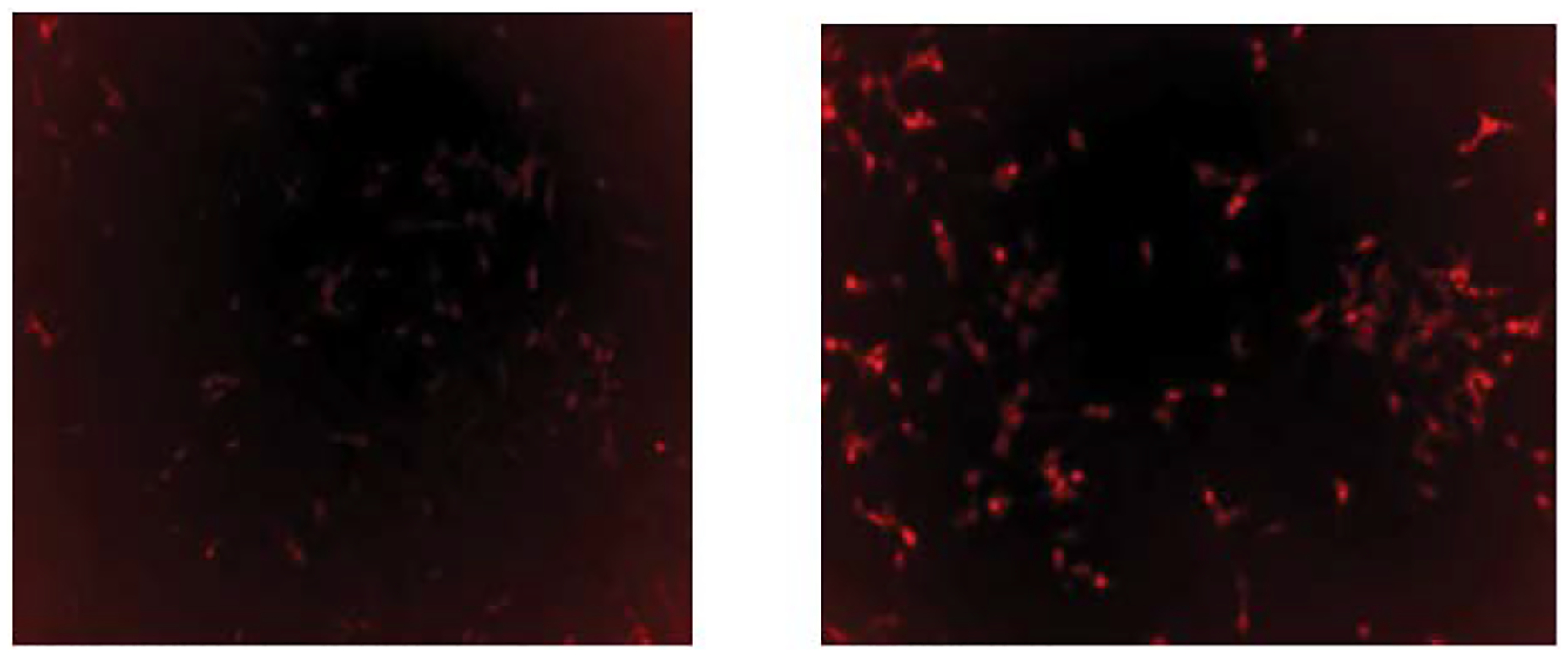
A) Fluroscence decreased due to Tubulin deploymerization in PR7 drug treated cells. B) Fluroscence increased due to Tubulin polymerization in DMSO vehicular control cancer cells.

**Figure 7:**
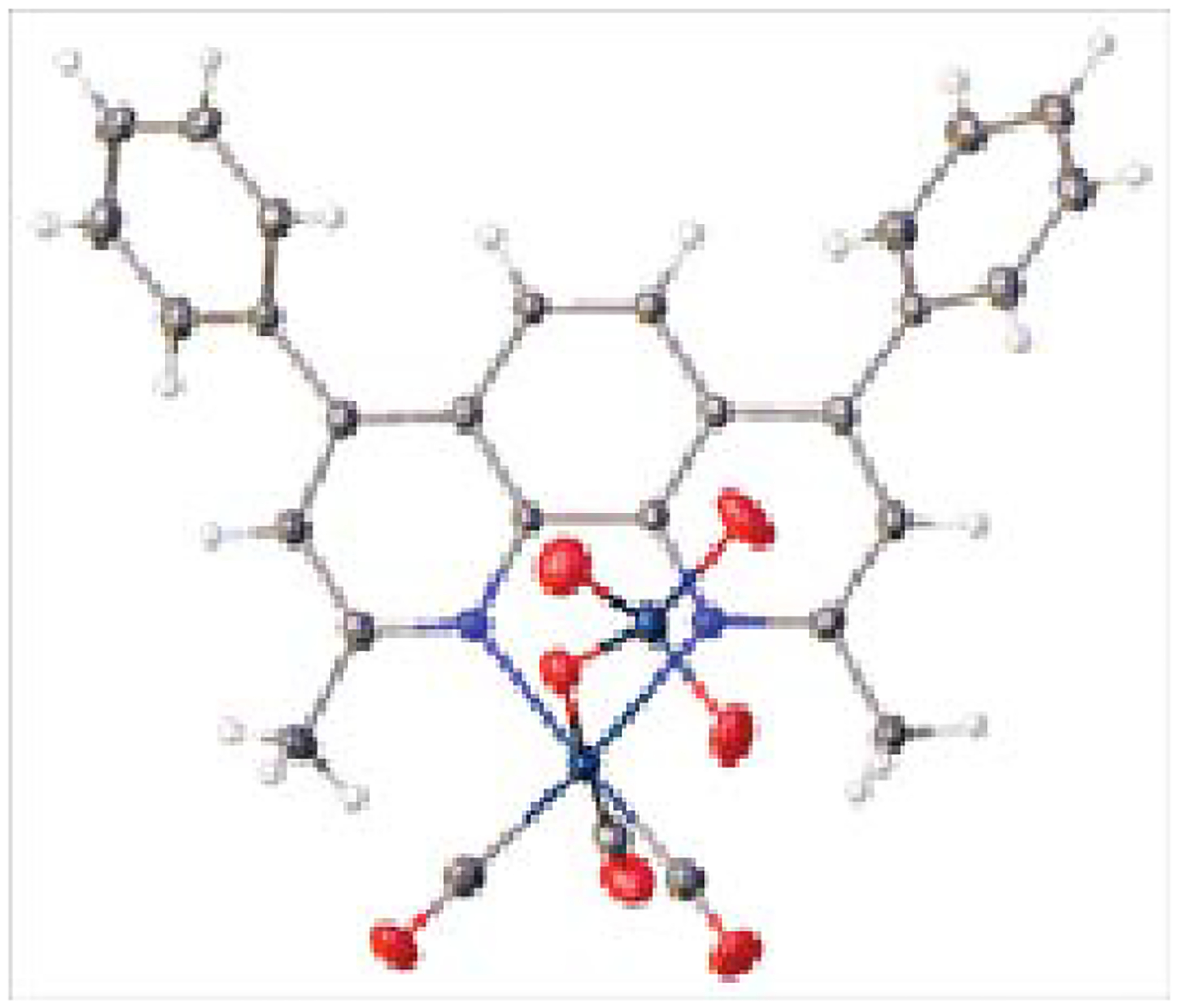
X-ray structure of PR7. The x-ray structure confirms the rhenium core, perrhenate, and ligand predicted from the line structure.

**Table 1:** Top molecules, canonical pathways and biofunctions from IPA results with their *p*-value in PR treated cells.

Top Canonical Pathways	P-Value	Overlap
*EIF2 Signaling*	1.23E-10	81.0 % (132/163)
*Sirtuin Signaling Pathway*	7.92E-08	75.5 % (148/196)
*Regulation of eIF4 and p70S6K Signaling*	1.18E-06	78.0 % (96/123)
*BAG2 Signaling Pathway*	3.08E-06	83.8 % (57/68)
*Ephrin Receptor Signaling*	2.42E-06	78.8 % (82/104)
Molecular and Cellular Functions		
	P-value Range	#Molecules
*Cell Cycle*	2.07E-02 – 2.07E-02	7
*Cellular Movement*	3.53E-02 – 2.52E-02	60
Top Tox Lists		
	P-value	#Molecules
*Mitochondrial Dysfunction*	7.61E-05	74.4 % 90/121
*Liver Necrosis/Cell Death*	5.04E-03	66.7 % 130/195
*Oxidative Stress*	9.07E-03	77.8 % 28/36
*NRF2-meddiated Oxidative Stress Response*	1.12E-02	67.4 % 91/135
*Acute Renal Failure Panel (Rat)*	1.13E-02	80.8 % 21/26
Top Upstream Regulators		
	P-value	Activation z-score
*NFE2L2*	3.31E-02	−1.039
*ANXA2*	6.00E-02	2.619
*AKT1*	3.67E-01	−2
*HDAC6*	3.69E-01	−2.213
*TGFB1*	3.82E-01	−2.454
